# Assessing the implementation and influence of policies that support research and innovation systems for health: the cases of Mozambique, Senegal, and Tanzania

**DOI:** 10.1186/s12961-015-0010-2

**Published:** 2015-04-18

**Authors:** Julius Mugwagwa, Daniel Edwards, Sylvia de Haan

**Affiliations:** Department of Engineering and Innovation, Development Policy and Practice Group, The Open University, Milton Keynes, MK7 6AA UK; The Council on Health Research for Development (COHRED), Route de Morillons 1-4, 1211 Geneva, Switzerland

**Keywords:** Health delivery, Mozambique, Policy implementation, Research and innovation system, Senegal, Tanzania

## Abstract

**Background:**

Without good policies it will be difficult to provide guidance to research and innovation systems. However, policies need to be followed through and implemented to have the desired effect. We studied the policies and strategies in place to support research and innovation systems for health in Mozambique, Senegal, and Tanzania, and looked at the extent to which these policies and strategies have been implemented.

**Methods:**

We reviewed documents and reports and conducted in-depth interviews with 16 key informants representing various actors of the national research for health systems.

**Results:**

The results illustrate that there are various policies and strategies governing research and innovation for health in the three countries. However, implementation of these policies and strategies is generally rated as being poor. The reasons highlighted for this include lack of policy coherence, lack of enforcement and accountability mechanisms, and a lack of financing for implementing the policies. These contextual factors seem to be of such importance that even the increased stakeholder involvement and political leadership, as mentioned by the interviewees, cannot guarantee policy implementation.

**Conclusions:**

We conclude that due to the contextual realities of the study countries, there is need for greater focus on policy implementation than on developing additional policies. Government institutions should play a central role in all stages of the policy process, and should ensure implementation of defined policies. Strong mechanisms, including financing, that strengthen the position and role of government in policy coordination and the oversight of the policy process will help increase efficient and impactful implementation of research and innovation for health policies.

## Background

The important roles played by research and innovation in health delivery cannot be over-emphasised. According to Prof Miriam Were of Kenya, ‘*health research* [should not] *only be about finding out what is new but also* [about] *ways of applying what is known and making use of available evidence*’ [1]. Policies are put in place to provide a framework for attainment of multiple and often competing socio-economic objectives. According to the research system for health framework [2] of the Council on Health Research for Development (COHRED), policies, priorities, and management make up three critical pillars of a research and innovation system. A number of authors agree that without good policies it will be difficult to provide guidance to any research and innovation system [3-5]. However, policies need to be followed through and implemented to have the desired effect. There is increasing academic, policy, and practice interest in how to attain such effective policy implementation [6].

Seeking to understand implementation of research and innovation for health policies requires an attempt to delineate and discuss what national research for health systems are. This helps to clarify important aspects of health policy implementation such as the scope of health, the individual and institutional stakeholders involved, the resources being allocated to the task, and the impact being made by policy implementation [4]. A policy defines a vision for the future, establishes targets and points of reference for the short and medium term, outlines priorities and the expected roles of different groups, and builds consensus and informs people [7].

This paper reports on a study that reviewed existing policies supporting research and innovation systems for health in Mozambique, Senegal, and Tanzania. The main research question for the study was ‘*what are the policies and strategies in place to support research and innovation systems for health in the selected countries and to what extent these have been implemented*’. The three countries’ involvement in the Research for Health Africa (R4HA) Programme, a programme focusing on strengthening research systems for health, implemented by COHRED and the New Partnership for Africa’s Development (NEPAD) Agency, made them good and convenient candidates for accessible, current, and relevant data on research and innovation systems for health which could be generalised to similar contexts, in an analytical rather than statistical sense. Further, the rationales for the inclusion of these countries in the R4HA programme are also appropriate bases for inclusion in this study. These were to include a diversity of experience and context through including a good spread of countries with different research for health systems (influenced through their respective colonial histories), and ensuring a good geographic spread by selecting countries with membership of three different Regional Economic Communities.

### Defining innovation and research systems

In this study, innovation is defined broadly as the creation and use of new, better, more effective, and more acceptable products, technologies, processes, and ideas [8]. We are also in agreement with neo-Schumpetarian thinking [9], which argues that systems of innovation do not emerge from industrialisation or technological advancement efforts only, but as Edquist [10] notes, from processes that are ‘*lengthy, interactive and social;* [and in which] *many people with different talents, skills and resources have to come together*’. Innovation systems require deliberate development and embedding within country-specific institutional and technological contexts [11,12].

The WHO defines a health research system as ‘*the people, institutions, and activities whose primary purpose is to generate high quality knowledge that can be used to promote, restore, and/or maintain the health status of populations. It can include the mechanisms adopted to encourage the utilization of research*’ [13]*.* The WHO framework for health research systems acknowledges that health research systems overlap with health systems and other research systems to varying extents depending on the context [14]*.* This is indeed the case in Mozambique, Senegal, and Tanzania, as shown in Table 1, and is taken into account when reviewing policies for research and innovation for health.

**Table 1:** **Policies that support research and innovation systems for health**

**Country**	**Policies and instruments dealing specifically with research and innovation for health**	**Other key policies and instruments with a bearing on research and innovation for health**	**Key international and national instruments influencing research and innovation for health systems**
Mozambique	Mozambique Science, Technology, and Innovation Strategy (2006)	National Information and Communications Technology (ICT) Policy (2002)	National constitution (1990)
			Millennium Development Goals (MDGs) Poverty Reduction Programme II (2006–2013)
	National Health Policy (2007)		
	Health Sector Strategic Plan (2007–2012)		
Senegal	National Health Development Plan (2009–2018)	National ICT Policy (2000)	National Constitution (2001) MDGs
		Economic and Social Policy Document (2011–2015)	
			Poverty Reduction Strategy Document (2010)
	Science and Technology Policy (2010)		
Tanzania	Health Sector Strategic Plan 2008–2015	National ICT Policy (2003)	National Constitution (1977)
	Tanzania National Health Policy (2012)	The National Road Map Strategic Plan To Accelerate Reduction of Maternal, Newborn and Child Deaths in Tanzania (2008–2015)	
		Human Resources for Health Strategic Plan (2008–2013)	
	National Science, Technology, and Innovation Policy (2012)		
			MDGs Primary Health Services Development Programme (2007–2017)
			Global Strategy and Plan of Action on Public Health, Innovation and Intellectual Property (2008)
			Tanzania Development Vision 2025
			National Strategy for Growth and Reduction of Poverty
			WHO Country Cooperation Strategy II (2010–2015)
			African Pharmaceutical Manufacturing Plan of Action

Source: Table developed by authors based on documents cited in the Table.

### Defining policy implementation

Policy implementation can be considered as the process of carrying out a government decision [6]*.* In defining policy implementation, many scholars have found it useful to make the conceptual distinction between the policy implementation process and policy outcomes, even though these are interactive in practice [15]. The implementation process involves action on behalf of the policy, whereas policy outcomes refer to the ultimate effect on the policy problem. Implementation is an iterative process in which ideas, expressed as policy, are transformed into behaviour, expressed as social action [6]*.* The social action transformed from the policy is typically aimed at social betterment and most frequently manifests as programmes, procedures, regulations, or practices.

Implementation has long been recognised as a distinct stage in the policy process, unique for representing the transformation of a policy idea or expectation to action aimed at remedying social problems [15]. Reflecting a process involving change over time, implementation is characterised by the actions of multiple levels of agencies, institutions, organisations, and their actors and is influenced by context throughout. As Parson [16] suggests, ‘*a study of implementation is a study of change: how change occurs, possibly how it may be induced*’.

Understanding the policy implementation process is important in part because many social programmes are publicly funded, and they are initiated and influenced by public policy. Assessing policy or programme implementation is also important for informing on-going decision making and exploring the extent of achievement of targets as well as how things can be done differently in more effective and impactful ways.

### Empirical gaps

There is a growing body of evidence on developing country research for health systems in general and African research for health systems in particular. For empirical insights in Africa see, for example, Kalua et al. [17], Moran et al. [18], African Union Commission – UNIDO [19], Berger et al. [20], Singer et al. [21], Mugabe [3], and the European and Developing Countries Clinical Trials Partnership [22], all of which, singly and collectively, make important contributions to the research and innovation for health knowledge base, particularly looking at the potential role of technologies and innovation in health systems and evidence-informed policymaking. They divert from the conceptual model of a linear process where evidence is generated, findings are made available, and eventually decisions are influenced. In reality, the process of evidence translation into decision-making within government or other institutions is rather more complex [5]. However, with a few exceptions, such as that of Mugabe [3], the majority of the studies and documents focus on the policymaking process itself. They study how policymaking is influenced by such a non-linear process characterised by negotiations among multiple actors, with their impact on knowledge uptake. This paper focuses specifically on the implementation of research and innovation for health policies unpacking, among others, the role of national and institutional contextual factors, policy content, and approaches. It does not seek to judge the content of the policies or propose alternative ones, but rather focuses on the process and context of successful policy implementation.

## Methods

We used both qualitative and quantitative methods to generate insight on policy availability and implementation and reviewed documents and reports. We gathered stakeholder insights, perceptions, and awareness of barriers to and facilitators of policy implementation through questionnaire-led interviews. Thematic analysis guided by themes emerging from the findings was employed for interrogating and analysing the data [23].

Between April and July 2014, a total of 34 questionnaires were emailed to the R4HA programme partners and other stakeholders who were not only a convenient sample, but are strategic and key actors in the health arena in the study countries. A mix of respondents was selected, representing actors from Government, regulatory agencies, and research (Table 2). These are people who are either tasked with policy implementation or directly affected by such implementation. The mix of interviewees enabled views of different aspects of the policy process to be received. The successful uptake or implementation of policies was based, rather than on key indicators, upon the perceptions and subjective experiences of this group of interviewees. Twenty-one respondents acknowledged receipt of the study questionnaire and expressed willingness to participate in the study. Finally, 16 respondents took part in the study, with 9 self-completing the questionnaire, while telephone or Skype interviews guided by the questionnaire were held with 7 respondents. The respondents’ diverse backgrounds and intimate involvement in research and innovation for health systems allowed engagement with and exploration of opinions and evidence on different aspects of the policy process. Table 2 below gives a breakdown of the respondent details.

**Table 2:** **Respondent details**

**Measure**	**Attribute**	**Mozambique**	**Senegal**	**Tanzania**
**Number**	**Total study respondents**	**6**	**4**	**6**
Years	Average length of service for respondents	10	12	15
Proportion (%)	**Time spent in different responsibilities**			
	Policy analysis	15	10	17
	Policy implementation	15	10	25
	Management	20	15	10
	Policymaking	40	30	35
	Other	10	35	13
**Number**	**Respondents and their category**			
	Government	3	3	3
	Funding agency	0	0	0
	Research	3	2	4
	Academic	2	2	3
	Manufacturing	0	0	0
	Regulatory agency	2	2	2
	Other	0	0	0
**Number**	**Respondents by categories** ^**a**^	10	9	12

Source: Table developed by authors.

^a^Some respondents fit into different categories hence higher count than total numbers of respondents per country.

The literature search targeted websites of organisations such as the WHO, World Bank, COHRED, UNESCO, NEPAD, the African Union, and specific government departments in the study countries in order to garner both broad and country-specific data on research and innovation for health systems. The search terms used included ‘health research’, ‘health innovation’, ‘health policy’, ‘policy implementation’, and ‘innovation policy’, and were used in combination with the countries and listed organisations.

## Results

The literature search resulted in a number of key documents on research for health and innovation in the three countries. A variety of policies and strategies were identified in areas such as industry, higher education, science, technology and innovation, and information and communications technology, all of which can have an impact on health. Table 1 lists the identified policies and strategy documents. The table also lists the national constitutions, which have a provision for the right to health, though the extent to which health is covered varies per country.

From the 16 in-depth interviews carried out across the three countries, two thirds of the respondents rated the status of implementation of all the key policies as ‘poor’ and implementation as the part of the policy process that receives ‘the least attention’. Reasons given include lack of adequate financial and human resource support; the reactive nature in which activities are implemented; limited attention to evaluation of the systems; information overload and asymmetries; and problems with managing overlaps and how to move from silos to systems. One respondent from Mozambique summed it up as:

‘*The weaknesses are actually in the entire process from problem identification to policy evaluation, but they become more visible at the implementation stage because that is where everything matters* … *that is where needs are met or not*’*.*

While some respondents felt the implementation problem was a result of poor policies and strategies being crafted in the first place, the general argument appears to be that, if there is adequate preparedness and facilitation to implement, better outcomes can be obtained. Better implementation of policies is more likely to have a bigger impact on other components of the process which as one respondent from Tanzania remarked:

‘*… have a tendency to remain good on paper. With implementation, things move from paper to practice, and you can’t hide that*’*.*

Across the three countries, the two areas that were said to receive the most attention (in terms of human, financial, and institutional resource allocation) are problem identification and policy formulation, perhaps partially, as one respondent from Mozambique put it ‘*because there is donor pressure for this to happen*’. Policy adoption was also rated as receiving a fair amount of attention. On the other hand, policy evaluation, like policy implementation, was said to be poor for reasons including lack of dedicated resources for policy evaluation and limited direct usage of results from evaluation activities. Half of the respondents identified this state of affairs as problematic, stating that accountability and transparency (trustworthiness, openness, and confidence in the systems) accruing from evaluation were significantly important for all stages of the policy process, including having the potential to stimulate better policy implementation. Meanwhile, the source of the resources for the different stages of the policy process was said to have a major bearing on the extent to which the policies got implemented. In Mozambique, resources from the government and other local sources were said to be more effective in implementation of long-term capacity building for both their health sector strategic plan and the science, technology, and innovation strategy than external donor resources.

As one respondent from Mozambique noted:

‘*Our experience is that money from the government is more useful for long term projects, while that from donors is good for short term projects. The R4HA funding is one example of short term funding coming in to fill gaps not covered by the government*’.

Contextual realities are important for policy implementation efforts. Interviewees were asked to review a number of key factors influencing policy development and implementation and indicate whether attention to these issues has stayed the same, improved, or worsened over the past 5 years. Table 3 shows, across the three countries, the opinion of the interviewees. The contextual issues relating to the policy process have at best remained the same, but in most cases, they have worsened.

**Table 3:** **Status of policy context issues in the last 5 years**

**Contextual issue**	**Whether issue has improved, stayed the same, or worsened in each country**		
	**Mozambique**	**Senegal**	**Tanzania**
**Allocated budget**	Same	Worsened	Worsened
**Stakeholder engagement**	Improved	Improved	Improved
**Political leadership**	Improved	Improved	Improved
**Policy coherence**	Worsened	Worsened	Worsened
**Enforcement and accountability mechanisms**	Worsened	Same	Worsened
**Incentives**	Same	Worsened	Worsened

Source: Table developed by authors from analysis of findings.

Stakeholder engagement and political leadership in policy processes is said to have improved over the last few years in all three countries, but as respondents from Senegal and Tanzania noted, this has not necessarily resulted in increased budget allocations. Mozambique was said to have managed to keep the budget fairly stable in the last 5 years due to external and private stakeholders funding of various aspects of the health sector strategic plan, which allowed government to free-up resources for research and innovation for health. Policy coherence (synergy and mutual reinforcement between policies) was said to have decreased over the same period. Perhaps this is not surprising given the large number of policies and other instruments relating to research and innovation for health as shown in Table 1. Stakeholder involvement in policymaking processes has increased. This is particularly true for the policy development stage, where across the three countries, government departments, external donors, academic institutions, civil society organisations, and even private companies appear to play active roles. The number of actors was said to decrease at stages such as financing, implementation, and evaluation of policies. Coordination and ensuring an even spread of players along the policy process were said to be weak, leading to incoherencies and dissipation of resources. While different organisations and people, including consultants, were said to play roles in coordination of implementation of policies, there was consensus that governments should be central in this so that alignment to overall national development goals can be ensured. According to one respondent from a government agency in Senegal:

‘*If we are to meet or exceed expectations in implementation of research and innovation policies for health (or any other policies), resources must be provided to strengthen government’s role in policy coordination and ability to hold actors in the policy arena to account*’*.*

In addition to strengthening of government policy oversight capacity, governments are also said to be in need of stakeholder support that is consistent and long-term. To this end, incentives to garner and sustain stakeholder support are needed, and they need to be continuously monitored to ensure their impact on policy implementation. There is a need to view and do things differently. As one respondent from Tanzania noted:

‘*We have heard a lot about the need for political will if policy implementation is to happen, but I would say, there is need for political skill as well. The will alone is not enough. We need different thinking*’.

Respondents alluded to a number of challenges that remain to be tackled for policy implementation to improve, and some lessons that have been learnt from concluded and on-going efforts. What stands out from the responses is the need to strengthen governments’ role in coordination of the policy process. There is a need to strengthen government institutions at various levels to enable them to perform this function. This also entails channelling resources through them, but as one respondent from Mozambique noted:

‘*Government departments need to assure donors, private sector, communities, and other stakeholders that they can be trusted to deliver efficiently and to utilise resources in a transparent manner*’.

## Discussion

The results show that implementation of research and innovation policies and strategies for health are influenced by institutional, sectoral, and national contexts. Three key themes emerge from the research: content, context, and approaches.

This study confirms that analysis of policy processes involves more than seeking an understanding of the mechanics of decision-making and implementation, but requires an unpacking of the underlying health priorities, policy content, and context, and the approaches to implementation of the policies [24].

The content, context, and approaches of the policy processes are products of issue framing, which is the way in which boundaries are drawn around problems, how policy problems are defined, and what is included and excluded. While problems and solutions related to health systems immediately lead to a focus around disease problems and proposed solutions through health delivery systems, the findings of this study confirm that the problems also encompass broader questions about health system organisation, and new forms of social, political, or economic arrangements [5]. Understanding and addressing these broader dynamics is necessary if policies for research and innovation in health are to be implemented effectively.

### Contextual factors

Health systems are complex and embedded in rules-based institutional arrangements, while sustained through social norms and informal practices [4]. Attitudes of the various individual and institutional players influence how policies for research and innovation for health will perform. This study confirms that, while research and innovation for health are becoming an area of increasing interest in the study countries, the main focus of the health sector is adoption and/or adaptation of research and innovation products from elsewhere [3]. This therefore means policies for research and innovation if not geared to promote innovation may not have much implementation or impact due to limited research and innovation happening in the first place. On the other hand, statements of intent to promote research and innovation as enshrined in some of the policy documents in Table 1 are only part of the story if there is no follow-up provision of resources and other mechanisms to direct the practice.

Related to the above, the contextual reality in the study countries is that institutions are partially functional and the ‘rules of the game’ are changing rapidly, including the rate of policy ‘turnover’ [4]. This results in some instances of the policy process being aborted or shifted unexpectedly. One respondent from Tanzania was emphatic on this:

‘*Policies are usually forgotten as people focus on projects, some of which have no relation at all to national development goals, but because they come with funding, these projects are implemented. Policies with no financing mechanisms will not go anywhere*’.

The above was also said to result from, or to be exacerbated by, the fact that the body of research and innovation for health knowledge is still emerging in the study countries, limiting their ability to predict the performance of policies and offer advice to policy and practice. Much of the evidence on the impact of alternative forms of health sector organisation (still) comes from advanced market economies, where institutional arrangements and behavioural norms are relatively stable [4].

### Content and approaches

This study confirms what policy scholars have established, namely that the policy process is political, from agenda setting to exploring possible problem resolution options, weighing up costs and benefits, making a rational choice about best options (decision-making), implementation, and evaluation [5,22,24]. ‘Evidence’ is called upon at any or all of these stages, but it was clear from this study that what constitutes good evidence for one level or one set of stakeholders is not necessarily the same for the other levels and stakeholders. For example, as one respondent from Senegal noted, ‘*local government and those in district hospitals and clinics tend to respond more to local constraints and support local innovations, while being sceptical of the relevance of ideas from the top*’. This study also challenges schools of thought which posit that the increased ability to generate and diffuse information rapidly through information technology will lead to emergence of a homogeneous knowledge economy [4]. The study shows that, while in the study countries there are communities of practice with overlapping understandings of (and roles in) research and innovation for health, their location on the policy process continuum has an overriding influence not only on the tools they deploy to implement policy, but what informs their view of the entire policy process. Input, perceptions, and voices of different stakeholders at various tiers are thus a critical part of the content for policies and approaches to implementing the policies. Related to this is the fact that the implementing officials are motivated by different individual, professional, and institutional factors, which need to be understood. There is therefore a need to understand the evidence which influences decision-making at each of these levels and, in addition, how the levels interact with each other [4,5].

## Conclusions

This paper addresses the status of policy implementation for research and innovation systems for health in three African countries. The research shows that Mozambique, Senegal, and Tanzania have a range of policies and strategies in place to support and guide research and innovation for health. However, implementation of these policies and strategies is generally rated poor. The reasons highlighted for this include lack of policy coherence, lack of enforcement and accountability mechanisms, and a lack of adequate financing for policy implementation.

We conclude that, due to the contextual realities of the study countries and many other low-income countries, as well as the rapidly evolving nature of the research and innovation for health terrain, there is a need to focus on the policy implementation process and not only on developing more policies. Government institutions should play a central role in all stages of the policy process and should ensure implementation of defined policies. Strong mechanisms, including financing, that strengthen the position and role of government in policy coordination and oversight of the policy process will help increase efficient and impactful implementation of research and innovation for health policies and strategies.

## Abbreviations

COHRED: Council on Health Research for Development
NEPAD: New Partnership for Africa’s Development
R4HA: Research for Health Africa
